# Alpha-ketoglutarate utilization in *Saccharomyces cerevisiae*: transport, compartmentation and catabolism

**DOI:** 10.1038/s41598-020-69178-6

**Published:** 2020-07-30

**Authors:** Jinrui Zhang, Bas Mees van den Herik, Sebastian Aljoscha Wahl

**Affiliations:** Department of Biotechnology, Delft University of Technology, van der Maasweg 9, 2629 HZ Delft, The Netherlands

**Keywords:** Applied microbiology, Industrial microbiology, Biochemical networks

## Abstract

α-Ketoglutarate (αKG) is a metabolite of the tricarboxylic acid cycle, important for biomass synthesis and a precursor for biotechnological products like 1,4-butanediol. In the eukaryote *Saccharomyces cerevisiae* αKG is present in different compartments. Compartmentation and (intra-)cellular transport could interfere with heterologous product pathways, generate futile cycles and reduce product yields. Batch and chemostat cultivations at low pH (≤ 5) showed that αKG can be transported, catabolized and used for biomass synthesis. The uptake mechanism of αKG was further investigated under αKG limited chemostat conditions at different pH (3, 4, 5, and 6). At very low pH (3, 4) there is a fraction of undissociated αKG that could diffuse over the periplasmic membrane. At pH 5 this fraction is very low, and the observed growth and residual concentration requires a permease/facilitated uptake mechanism of the mono-dissociated form of αKG. Consumption of αKG under mixed substrate conditions was only observed for low glucose concentrations in chemostat cultivations, suggesting that the putative αKG transporter is repressed by glucose. Fully ^13^C-labeled αKG was introduced as a tracer during a glucose/αKG co-feeding chemostat to trace αKG transport and utilization. The measured ^13^C enrichments suggest the major part of the consumed ^13^C αKG was used for the synthesis of glutamate, and the remainder was transported into the mitochondria and fully oxidized. There was no enrichment observed in glycolytic intermediates, suggesting that there was no gluconeogenic activity under the co-feeding conditions. ^13^C based flux analysis suggests that the intracellular transport is bi-directional, i.e. there is a fast exchange between the cytosol and mitochondria. The model further estimates that most intracellular αKG (88%) was present in the cytosol. Using literature reported volume fractions, the mitochondria/cytosol concentration ratio was 1.33. Such ratio will not require energy investment for transport towards the mitochondria (based on thermodynamic driving forces calculated with literature pH values). Growth on αKG as sole carbon source was observed, suggesting that *S. cerevisiae* is not fully Krebs-negative. Using ^13^C tracing and modelling the intracellular use of αKG under co-feeding conditions showed a link with biomass synthesis, transport into the mitochondria and catabolism. For the engineering of strains that use cytosolic αKG as precursor, both observed sinks should be minimized to increase the putative yields.

## Introduction

α-Ketoglutarate (αKG, 2-oxoglutarate, 2-oxoglutaric acid) is a key tricarboxylic acid (TCA) cycle intermediate and an important intermediate of many catabolic and anabolic processes. The number of metabolic enzymes known to be regulated by αKG levels has increased significantly in recent years and αKG emerged as a ‘master regulator metabolite’, as reviewed in^1^. αKG is a high-value organic acid that is reported to extend the lifespan of adult *Caenorhabditis elegans*^2^. αKG supplementation was shown to increase oxidative stress resistance and enhances freeze–thaw tolerance of the yeast *Saccharomyces cerevisiae*^3^. It has broad industrial application possibilities, i.e. it is a precursor for the synthesis of dietary supplements, pharmaceuticals, cosmetics, and heterocyclic compounds^4–6^. In practice, this means that αKG can be used as a biochemical building block for the production of relevant chemicals, e.g. 1,4-butanediol, l-ornithine, l-glutamate^7,8^.

Chemical synthesis of αKG is possible through various routes, but it always is a multi-step, non-environmentally friendly, route partly involving toxic chemicals^4,9^. Therefore, more attention is being directed to the more sustainable microbial production of αKG^5,10,11^. Natural producers include several fungi and bacteria as well as oleaginous yeast *Yarrowia lipolytica*. In *Y. lipolytica* titers of 97 g/L with a production rate of 0.047 g/g/h are reported under small scale conditions. No examples of industrial scale titres are present in literature to the best of our knowledge. Due to the lack of current industrial production capacity of αKG and the known capability of *S. cerevisiae* to produce high titers of organic acids (100 g/L Succinic Acid, Pyruvate, Lactic Acid), *S. cerevisiae* is an attractive cell factory to consider for αKG production.

One successful example of using *S. cerevisiae* generated a high supply of the precursor αKG to enable high-level production of l-ornithine that can be used as precursor metabolite for a range of relevant natural products^12^. The applied modular pathway rewiring strategy involved rewiring of the urea cycle, subcellular trafficking engineering and pathway re-localization. Such approach was only possible with detailed knowledge of the metabolic networks and their regulation. In particular, when aiming for the production of αKG and its derivatives, enhancement of the precursor supply is a very common strategy to increase the flux towards the desired amino acids^12,13^. Learnings from the modular pathway rewiring approach in^12^ also included the uncertainty of the role of mitochondrial carriers, stressing the importance of understanding intracellular transport. For example, Wahl et al*.*^14^ showed that compartmentation reduced the production yield of succinic acid in engineered *S. cerevisiae* strains. In short, the compartmentation of metabolites in different subcellular organelles, mainly the cytosol and mitochondria can interfere with metabolic engineering strategies^15–23^.

*Saccharomyces cerevisiae* has been classified as a Krebs-negative species—i.e. no growth was observed using TCA cycle intermediates as carbon sources^24,25^. Surprisingly, in 1935 Krebs^26^ showed that *S. cerevisiae* showed an increase in oxygen uptake when αKG was used as sole carbon source, but these results were not mentioned in later reviews^24^ and *S. cerevisiae* was assumed to be unable to grow on any TCA-cycle intermediate as sole carbon source. While growth was not observed, several TCA cycle intermediates were shown to be catabolized under specific conditions, such as co-consumption with glucose^14,24,27,28^. Other strains than the often-used CEN.PK 113-7D strain were also reported unable to grow on dicarboxylic acids, for example Rodriguez and Thornton^29^ reported the inability of *S. cerevisiae* MD26 to grow on malate.

In this work, systematic experiments to identify the αKG uptake mechanism and resulting physiology were performed. Especially, batch and chemostat cultivations under varying extracellular pH and substrate concentrations were used. Secondly, the metabolism of αKG was studied using tracer experiments.

## Results

To study the αKG uptake mechanism and growth physiology using αKG as sole carbon source, batch and chemostat cultivations were performed at different substrate concentrations as well as different pH. Our observations show that *S. cerevisiae* can grow on αKG as sole carbon source under several pH conditions and a broad concentration range. An overview of the experimental design and observations is given in Table 1.

**Table 1:** Experimental conditions and physiological observations for growth on αKG as sole-carbon source and co-mixture with glucose in batch and chemostat conditions. More details on the batch results can be found in Fig. 1 and Supplementary Figure S2.

Growth mode	C-Source	pH	Growth?	Observations
Batch	2 g/L αKG	2.5	Yes	Growth is observed using αKG between pH 2.5 to 5. Both H_2_αKG and HαKG must be transported to explain obtained growth results
		3	Yes	
		4	Yes	
		5	Yes	
		6	No	
	2.5 g/L αKG	4	Yes	A growth rate of 0.087 ± 0.002 h^−1^. No decrease of growth rate for different concentrations of αKG
	5 g/L αKG		Yes	
	10 g/L αKG		Yes	
	20 g/L Glucose 2.8 g/L αKG	4	Yes	Maximum growth rate using glucose and αKG is µ = 0.40 h^−1^. αKG is not consumed while the glucose concentration is high, nor does αKG inhibit growth
Chemostat	10 g/L αKG	3	Yes	Residual αKG concentrations range from 30.1 to 37.9 mmol/L. The αKG uptake rate and residual αKG concentration were similar for the three different pH conditions
		4	Yes	
		5	Yes	
		6	No	
	20 g/L Glucose 2.8 g/L αKG	4	Yes	Both residual αKG (0.98 ± 0.28 mmol/L) and glucose (0.34 ± 0.01 mmol/L) are low, indicating that under continuous conditions co-consumption does also occur

### *Saccharomyces cerevisiae* can use αKG as carbon source: pH dependency and co-feed with glucose in batch cultivations

One hypothesis for the assumed disability of *S. cerevisiae* to grow using αKG as sole carbon source could be the inability to import αKG. Generating conditions, in which membrane diffusion of αKG is enabled, are low pH conditions (pK_a1_ = 2.7, pK_a2_ = 4.6). In batch cultivations with αKG as sole carbon source, growth was observed between pH 2.5 and pH 5 (Figure S2). This pH range suggests that there is growth not only when passive diffusion over the membrane is possible (pH < 4, H_2_αKG present, Figure S1), but also when there is basically no H_2_αKG, indicating an additional HαKG^−^ transport mechanism.

Using different αKG concentrations at pH 3, the maximum growth rates and biomass yields in co-cultivation were found to be comparable at 0.087 ± 0.002 h^−1^, and 0.37 ± 0.01 gDW/gαKG, respectively (Figure S2). This showed that higher concentrations of the acid did not cause inefficiency due to futile cycling over the membrane, which was expected with high concentrations of acid at low pH. This indicates that the periplasmic-membrane of *S. cerevisiae* has a low permeability for H_2_αKG, as a higher driving force (larger EC/IC concentration differences imposed by different extracellular concentrations) did not lead to futile cycling at concentrations ranging from 2 to 10 g/L.

Transport of αKG over the periplasmic-membrane seems highly pH dependent. Growth is observed only at low pH (≤ 5), indicating that the (fully) protonated acid is diffusing over the membrane. At pH 5, HαKG^−^ was the major species present, and almost no (< 0.1%, 0.027 mM) H_2_αKG was present. For H_2_αKG uptake at pH 5 (16.25 mmol/Cmol/h) to be fully driven by passive uptake, a permeability coefficient of 16.25/0.027 = 603 L/Cmol/h (6.34 μL/gDW/s) would be needed. Such value is much higher than previously reported for a similar acid (malate) by^27^ (permeability coefficient of 0.1 μL/gDW/s). Thus, it seems unlikely that all αKG import originates from H_2_αKG diffusion at pH 5. While charged HαKG^−^ should not able to pass the membrane by diffusion, the observed growth suggests that there must be a transporter protein/permease that facilitates transport of the mono-dissociated species via an unknown mechanism. No growth is observed for pH ≥ 6, which indicates that fully dissociated αKG cannot be transported at rates sufficient for growth. Camasara et al.^30^ found that HαKG was (weakly) transported by MAE1p (glucose repressed malate transporter) expressed in *S. cerevisiae*. Nevertheless, this physiological evidence of a transporter of the mono-dissociated species of the dicarboxylic acid αKG was not reported in the literature before to our best knowledge^24,28^.

**Figure 1:**
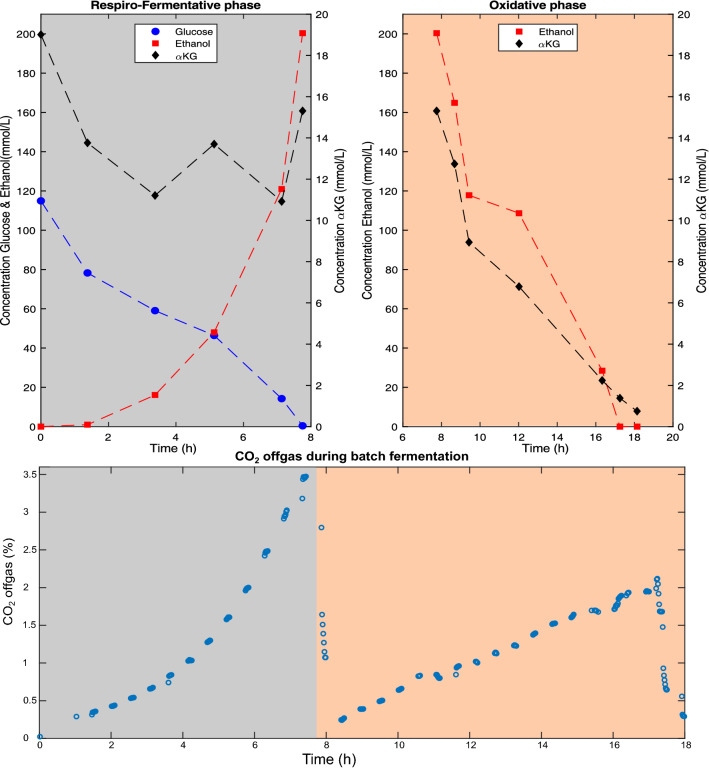
Time course of glucose, ethanol, αKG (upper panels) and CO_2_ (lower panel) during the bioreactor batch cultivation (pH 5) using glucose and αKG as substrates. The cultivation profile is split in two phases based on the (1) glucose uptake and ethanol production (respire-fermentative phase) and (2) consumption of ethanol together with αKG (oxidative phase).

The ability of co-consumption of αKG with glucose and ethanol was investigated at pH 5 by measuring extracellular concentrations of αKG, glucose and ethanol during batch cultivation (Fig. 1). The results showed that glucose was consumed first while ethanol and minor amounts of acetate (1.8 mM at the end of the respiro-fermentative phase) were produced. No αKG consumption during the respiro-fermentative growth phase was observed. After glucose depletion, ethanol and αKG were consumed simultaneously. Nonetheless, when αKG is present as C-source in the presence of glucose, it could be used as a precursor for glutamate synthesis. The lack of αKG consumption was therefore most likely caused by the absence of αKG import, putatively caused by glucose repression^31^.

These results show that (1) *S. cerevisiae* can grow on αKG as sole carbon source, (2) the utilization of αKG as carbon source is dependent on extracellular pH, and (3) uptake at pH 5 must be facilitated by a HαKG-transporter. The earlier categorization as Krebs-negative phenotype, was based on limited cultivation conditions and should be revised.

### Physiological characteristics for αKG as carbon source in chemostat cultivations

To further analyze the transport and catabolism of αKG, chemostat cultivations were performed using either only αKG or a mixture of glucose and αKG (Table 2). The αKG uptake rates and residual αKG concentrations were similar for the three different pH cultivations, indicating that the uptake rate of αKG was the limiting factor under these conditions. During co-consumption with glucose the residual concentration of αKG was lower compared to αKG only feeding. This is consistent with the lower αKG uptake flux (136 vs 600 μmol/gDW/h), nevertheless, the difference in residual concentration is very high (1 vs 33 mM) suggesting that that the transport mechanism and/or metabolism could be different. In contrast to the batch conditions, there is co-consumption of glucose and αKG, probably due to the low residual glucose concentration.

**Table 2:** Biomass specific rates and residual concentrations for the chemostat experiments with αKG (and glucose) feeding. Rates are in μmol/gDW/h. Rates for αKG/glucose co-feed were averages of 3 independent cultivations.

Carbon source(s)	pH	q_αkg_ (μmol/gDW/h)	q_glc_ (μmol/gDW/h)	µ (h^−1^)	Biomass concentration (g)	Residual αKG (mM)	Residual glucose (mM)
10 g/L αKG	3	554.9	–	0.028	1.56 ± 0.08	37.92	–
	4	615.8	–	0.027	1.64 ± 0.03	30.11	–
	5	578.3	–	0.027	1.58 ± 0.02	33.67	–
2.8 g/L αKG 20 g/L glucose	4	136.6 ± 6.8	1,054.9 ± 30.7	0.101 ± 0.002	11.59 ± 0.35	0.98 ± 0.28	0.34 ± 0.01

Intracellular metabolite concentrations under carbon limited chemostat conditions for αKG as sole carbon source, glucose and αKG co-feeding, as well as glucose only were measured and compared (Table 3). Especially using αKG as sole carbon source triggered significant differences in the metabolome compared to the other two conditions. The concentrations of TCA-cycle intermediates following αKG (Fum, Mal) were elevated, whereas TCA-cycle intermediates preceding αKG (Cit, iCit) were lower. The glycolytic intermediates were at lower concentrations for growth on αKG as sole carbon source. The differences between co-feeding and glucose only conditions were less pronounced. There was a slight increase in all intermediates concentrations during co-feeding.

**Table 3:** Steady state intracellular metabolite concentrations (μmol/gDW) for different cultivation conditions: αKG as sole carbon source, αKG and glucose co-feeding, and glucose only. PEP, Pyr, Succ and Glu were not measured for αKG only cultivation.

**Condition**	G6P	Pyr	Cit	iCit	αKG	Succ	Fum	Mal	Glu
αKG	1.14 ± 0.02	–	3.34 ± 0.09	0.11 ± 0.00	22.21 ± 7.70	–	4.16 ± 0.24	15.50 ± 1.02	–
αKG and glucose	8.37 ± 0.20	0.33 ± 0.08	7.89 ± 0.39	0.17 ± 0.02	2.81 ± 0.35	1.49 ± 0.16	0.87 ± 0.10	4.00 ± 0.05	116.26
Glucose (taken from^32^)	5.42 ± 0.15	2.20 ± 0.02	6.96 ± 0.15	0.40 ± 0.02	1.82 ± 0.04	0.96 ± 0.01	0.67 ± 0.01	3.01 ± 0.05	104.24 ± 1.51

### αKG compartmentation and flux distribution based on ^13^C-tracer experiments

The observations from the chemostat cultivations showed that there is co-consumption of αKG and glucose under low glucose concentrations. This co-consumption can be exploited to introduce a tracer, ^13^C αKG that will allow to study (intracellular) transport and catabolism. The introduction of the co-substrate had a limited impact on the intracellular metabolome. Especially metabolites of the TCA cycle remain at comparable concentrations for the two different substrate feeding conditions (see Table 3), which was not the case for growth on only αKG. At steady-state ^13^C-labelled αKG was added using the BioScope^33^. From the experimental design, ± 80% labelled, extracellular, αKG (total carbon) was expected, which was confirmed in the experiment (measured enrichment 78.5%).

A steep increase in intracellular αKG enrichment was observed in the first seconds after introducing labelled αKG. Subsequent and slow incorporation of the labelled carbon in glutamate and TCA-cycle intermediates confirms the use of exogenically introduced αKG for both biosynthesis and energy generation. No increase in enrichment was observed in glycolytic or PPP metabolites, showing that there was no gluconeogenic activity. The inflow of un-labelled glucose is larger than the uptake of labelled αKG, which leads to a low total enrichment of metabolites.

The enrichment data was used for the estimation of fluxes, with focus on fluxes around αKG transport and utilization. A metabolic model (Table S1) was used to determine the intracellular transport between cytosol and mitochondria, including oxidation in the mitochondria and utilization for biosynthesis (glutamate synthesis) in the cytosol. Using this approach, the percentage of αKG present in the cytosol and mitochondria was also determined. To analyse if compartmentation of αKG is required to explain the experimental data, labelling patterns were also simulated without the introduction of αKG compartmentation.

**Figure 2:**
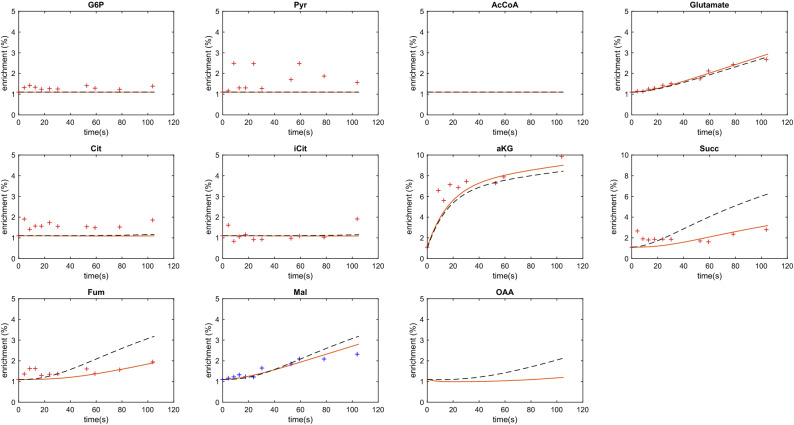
Enrichment measurements ( +) and simulations for stationary metabolism under glucose, αKG co-feeding conditions. Solid lines represent the simulation using a model with compartmentation of αKG. Dashed lines represent the results when using a model without compartmentation of αKG. The malate enrichment was adjusted compared to the original measurements as discussed in the methods (shown in blue).

Figure 2 shows the measured, C-molar, enrichment patterns and the simulated patterns after parameter estimation for both scenarios. The simulation including αKG compartmentation reproduced the trends of the labelling measurements well. I.e. the simulated glutamate and fumarate enrichments show a similar increase over time as the measurements. Neglecting αKG compartmentation leads to significantly higher deviations from the experimental measurements (Fig. 2, dashed lines), supporting that αKG compartmentation is structurally needed to reproduce the measured enrichments of TCA-intermediates. Especially, the slow rate of label incorporation into (mitochondrial) metabolites downstream of αKG (fumarate, succinate as well as malate) was only reproduced when compartmentation was included. Additionally, the enrichment transients of glutamate and αKG increase slightly and match the measured enrichments more accurately.

The transport mechanism could not be identified from the obtained enrichment data and modelling. Reported were antiport mechanisms: Citrate/αKG^34^ as well as malate/αKG^35^. Because of the slow enrichment in Citrate as well as malate, no conclusions about these antiporters could be derived.

**Figure 3:**
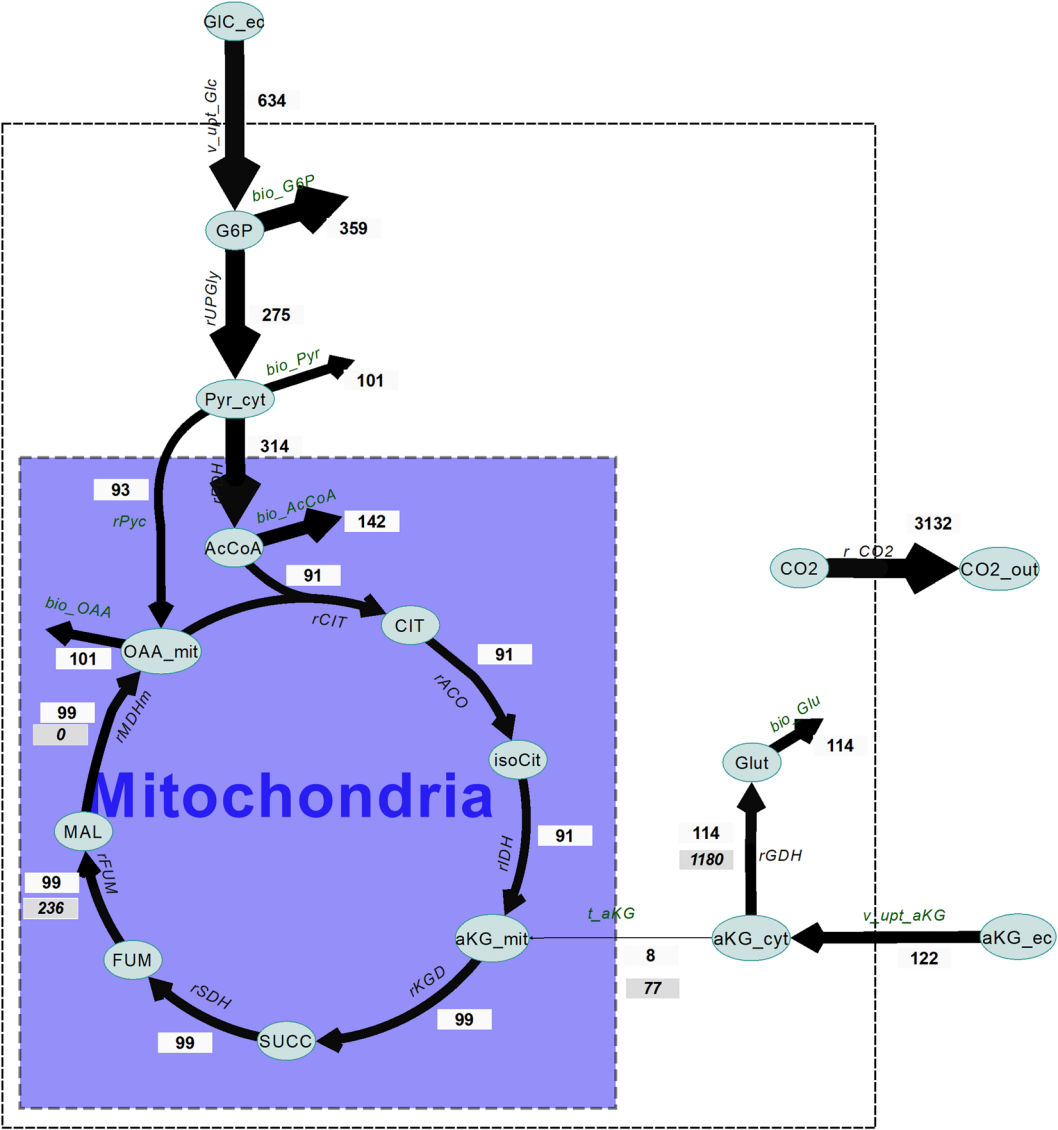
Estimated flux values shown for the used network**.** Flux values are given in μmol/gDW/h and listed in Table S2. Fluxes are categorized into 5 ranges (0–2, 2–10, 10–50, 50–500 and 500 above). The net flux values are shown.

The obtained flux distribution around αKG (Fig. 3) gave a comprehensive overview of the usage of αKG under co-feed conditions. It was found that 94% of the introduced αKG was used for the synthesis of glutamate (biosynthesis), the rest was transported into the mitochondria and fully oxidized. This observation was dependent on the used biomass equation and can also be obtained from a flux balance analysis, only using extracellular production and consumption rates (Figure S4). However, using labelled αKG also allowed to determine the exchange fluxes between cytosol and mitochondria and between αKG and glutamate. The calculated exchange fluxes were high in comparison to the net fluxes, indicating a high bidirectionality of the reactions. Glutamate is the major sink of imported αKG, and the large intracellular glutamate pool buffers the ^13^C-tracer gradient, leading to slower enrichment transients.

The model includes a parameter representing the ratio of cytosolic/mitochondrial αKG that was also estimated using the experimental data. The fraction of cytosolic αKG was determined to be 88 ± 1%. This shows that the majority αKG is cytosolic, which is beneficial for the introduction of a cytosolic production pathway. The cytosolic fraction can be used to calculate concentrations (assuming a volume distribution of 7% mitochondrial and 70% cytosolic^36^) in the cytosol (5.29 mM) and mitochondria (7.01 mM), equal to a concentration ratio between the mitochondria and cytosol of 1.33. A thermodynamic study of mitochondrial transporters by^14^ showed that when assuming Mal/Pi transport in equilibrium, the pH difference between the mitochondria and the cytosol can generate a concentration ratio for αKG mit/cyt of 9.96. The obtained ratios are consistent w.r.t. to flux direction and thermodynamics, i.e. the TCA-cycle has a negative ΔG in the oxidative direction (Table S5).

## Discussion

It was found that *S. cerevisiae* can utilize and grow using αKG as sole carbon source, suggesting that the Krebs-negative classification should be reconsidered. The classification was based on growth conditions that did limit the transport of αKG. At pH ≤ 5, transport was present and growth was observed. Interestingly, the experiments performed by^27,28^ showed that growth on fumarate as sole carbon source was not possible at similar, low pH, although transport was feasible. Additionally, catabolism of fumarate was observed under co-consumption conditions with glucose. One main difference between αKG and fumarate as substrates is a lower requirement of anaplerotic reactions as αKG for glutamate synthesis is readily available. This hypothesis could be tested by labelling experiments using αKG as sole carbon source and laboratory evolution experiments with decreasing glucose/fumarate ratios to eventually obtain growth on fumarate only as well.

The experimental design of the ^13^C-tracer experiment was based on the experimental design from Wahl et al.^14^ and the observed co-consumption of αKG and glucose. In the case of succinic acid in the medium a high simultaneous im- and export was observed. For αKG this was much lower leading to slower enrichment gradients during the timespan of the experiment. With slow gradients, less information for flux identification is available and the used model was reduced accordingly. I.e. not all bidirectional transporters and reactions could be identified and were not included in the model. Nevertheless, compartmentation of αKG and the exchange fluxes with glutamate could be determined from the tracer experiment.

The observed co-consumption could in principle also be explained by sub-populations as observed for several other substrate conditions^37–39^: One consuming only glucose, and the other only consuming αKG. A population using only αKG would require gluconeogenesis to produce relevant biomass precursors. Under such conditions, labelling will be observed in gluconeogenetic intermediates, which was not found here.

The introduction of αKG co-feeding increased the intracellular αKG concentration (from 1.8 to 2.8 µmol/gDW) and potentially influenced the intracellular distribution of αKG between cytosol and mitochondria, thus the results for compartmentation might be condition specific. On the other hand, the concentration of up- and downstream metabolites was comparable to glucose only conditions, suggesting that metabolism was not significantly altered.

## Conclusions

The study analyzed the fate of cytosolic αKG in *S. cerevisiae*. This intermediate is a relevant precursor for different product pathways like 1,4 butandiol (BDO) or gamma-butyrolactone (GBL). Using co-feeding of glucose and ^13^C labeled αKG intracellular consumption was observed for biomass synthesis, but also transport into the mitochondria and full oxidation. These sinks should be taken into account when engineering strains with cytosolic αKG as precursor.

Furthermore, it was found that the cytosolic αKG can serve as sole carbon source for growth. This was not expected as *S. cerevisiae* was classified Krebs negative. Most probably, earlier studies were performed in a pH range with limited αKG transport over the periplasm membrane that was insufficient for observable biomass synthesis. In future studies, it would be beneficial to include a broad range of conditions, especially for substrates where transport could depend on environmental conditions (like pH).

## Materials and methods

The wildtype strain *S. cerevisiae* CEN.PK 113‐7D (Fungal Biodiversity Center, Utrecht, the Netherlands) was used in this work, stocks were kept in glycerol at − 80 °C.

### Batch cultivation

Minimal media was used for aerobic batch cultivations consisting of demineralized water, 5.0 g/L (NH_4_)_2_SO_4_, 3.0 g/L KH_2_PO_4_, 0.5 g/L MgSO_4_·7H_2_O, vitamins (1 mL/L) and trace elements (1 mL/L) compositions were similar to^40^. Cultivations with different pH values (3–6) were performed using 2 to 10 g/L αKG as sole carbon source, the pH was adjusted using 2 M KOH and 2 M HCl. All cultivations were performed in 200 mL shake flasks with 100 mL working volume, pH was measured during the whole experiment to ensure no large changes occurred in experimental conditions. The optical density (OD 660 nm) was measured using a spectrophotometer (Biochrom Libra, UK). The extracellular concentrations were measured using HPLC as reported earlier^14^.

### pH step chemostat experiment

Aerobic chemostat cultures were performed using minimal medium consisting of demineralized water, 0.5 g/L (NH_4_)_2_SO_4_, 5 g/L NH_4_H_2_PO_4_, 0.5 g/L (NH_4_)_2_HPO_4_, 0.8 g/L MgSO_4_·7H_2_O, vitamins (1 mL/L) and trace elements (1 mL/L) compositions were similar to^40^ and αKG (10 g/L) was the sole carbon source. After the batch phase, a carbon-limited chemostat (D = 0.025 h^−1^) was started at pH = 3 in a 2 L bioreactor (Applikon Biotechnology B.V., Delft, the Netherlands) with a working volume of 1 L. After steady-state was reached, the feed medium was replaced by identical medium, with a different pH. pH was kept constant at the respective value using 2 M KOH. With each step, the pH was increased by 1 unit until biomass washout was observed. For each step, steady-state was assumed to be reached after 5 residence times and a stable CO_2_ concentration in the off-gas. The temperature was controlled at 30 °C and the head space overpressure was kept at 0.3 bar, aeration rate was 0.5 vvm and the stirrer speed was set at 600 rpm to assure aerobic conditions. At steady-state, intracellular, extracellular and biomass samples were taken. Biomass concentration was measured using 10 mL samples, which were filtered over a pre-weighed and pre-dried filter (0.2 μm, Waters). Samples were dried in a 70 °C oven until a stable weight was reached. Extracellular concentrations were measured using HPLC, and intracellular concentrations were processed and analyzed as in^14^.

### ^13^C tracer experiments using the BioScope plug-flow reactor

**Figure 4:**
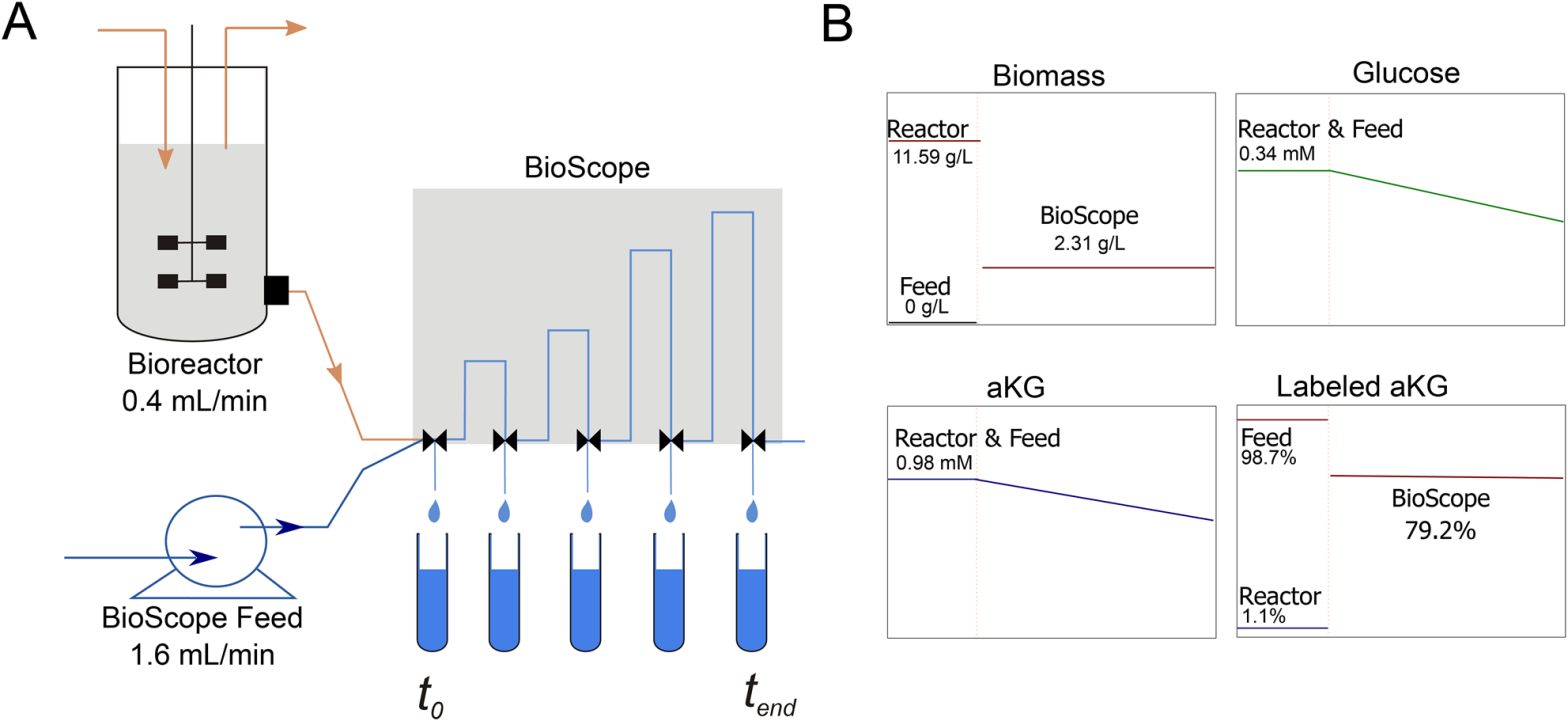
(**A**) Schematic overview of the BioScope labeling experiment^33^ and (**B**) Experimental design for concentration and enrichment time course (adapted from^14^). The BioScope has inflows from the bioreactor (flow 0.4 mL/min) and the feed (1.6 mL/min). The BioScope feed contains labeled αKG matching the concentration of αKG of the bioreactor broth. The feed also matches the residual glucose concent.

To obtain high concentrations of biomass for BioScope^33^ experiments, αKG co-consumption with glucose was performed, using 2.8 g/L αKG and 20 g/L glucose (0.126 Cmol αKG/Cmol glucose). Glucose and αKG co-feed was first studied in a batch and chemostat cultivation to characterize the co-consumption (rates, growth and residual concentrations) by measuring biomass dry weight and extracellular concentrations by HPLC during the batch phase and chemostat steady-state. The aim of the continuous BioScope labeling experiments was to achieve a fast-labeling gradient in the extracellular space without disturbing the metabolic steady-state. The BioScope was connected to the bioreactor with a broth inflow rate of 0.4 L/min, and a 1.6 mL/min flow rate for the feed solution of [U-^13^C5] αKG (custom-synthesized by Sigma-Aldrich BV, the Netherlands) and unlabeled glucose to reach 5 × dilution of the broth (Fig. 4A). A recycle loop system was used to prevent air bubbles entering the BioScope and to lower the transport time between the bioreactor and the BioScope. The concentrations of unlabeled glucose and ^13^C labeled αKG in the feed were the same as the extracellular concentrations, to avoid changes in extracellular concentrations after the dilution. Two similar experiments were performed: (1) using unlabeled αKG and unlabeled glucose in the feed media for intracellular concentration measurements, and (2) using [U-^13^C5] αKG and unlabeled glucose in the feed media for ^13^C tracing, allowing for immediate labeling with about 80% enrichment without perturbing steady-state (Fig. 4B). To mimic the flow of 0.5 vvm in the bioreactor a flow rate of 0.086 L/min was used to pass through the gas channel of the BioScope^33^.

### Intracellular and extracellular sampling and sample analysis

The quenching, extraction and analysis of the metabolites were performed similar to the methods in^14^. In brief, the sampling times for BioScope experiment were at 0, 4.3, 8.6, 12.8, 17.6, 23.9, 30.3, 41.5, 52.7, 78.2, and 103.7 s. Intracellular samples from the BioScope were collected for about 36 s (~ 1.2 mL) at each port. 120 μL of ^13^C cell extract were added as internal standard for concentration measurements samples. The procedure was similar for mass isotopomer enrichments samples with the exception that no ^13^C cell extract was added.

Around 1 mL extracellular sample from each port was obtained by vacuum filtration^14^. To determine the concentration in the extracellular space, 100 µL of the filtrate and 20 µL of ^13^C cell extract as internal standard were mixed and processed comparable to intracellular samples.

### Metabolic network construction, FBA and ^13^C-MFA

A stoichiometric and atom transitions metabolic model was constructed to analyse the metabolism of αKG (and glucose). The model included a full, oxidative TCA-cycle located in the mitochondria, lumped glycolysis and glutamate synthesis. Furthermore, αKG transport between the mitochondria, cytosol and extracellular space was included (Table S1). In total the model included 12 intracellular metabolites and 24 fluxes of which 4 were bi-directional.

### Flux balance analysis (FBA)

A FBA using the metabolic network as described above was performed to estimate the distribution of intracellular fluxes under fixed experimental conditions, with biomass as objective function, measured extracellular rates (*μ*, *q*_*αKG*_, *q*_*glc*_, *q*_*CO2*_) were constrained. Furthermore, inequality constraints were set for irreversible fluxes. As a final constraint, the maintenance coefficient is set at 0, to calculate theoretically optimal fluxes. To solve the optimization problem shown below, the function linprog was used in MATLAB 2018a.

$$v_{opt} = arg\max v_{\mu } { }subject{ }to{ }\left\{ {\begin{array}{*{20}l} {Nv = 0} \\ {v_{opt,meas} = q_{meas} } \\ {v_{maintenance} = 0} \\ {v_{irr} \ge 0} \\ \end{array} } \right.$$

### ^13^C-metabolic flux analysis (^13^C-MFA)

For the flux estimation (^13^C-MFA) using the ^13^C-enrichement data several assumptions were made:

I. In the BioScope experiment, the residual substrate concentrations of αKG was lower than previously measured concentrations of extracellular metabolites^14,33^, leading to less accurate measurements and rate determination. Nonetheless, the calculated slopes indicate similar uptake rates for glucose. Combined with the usage of a recycle loop system as described by^33^, similar conditions are expected in the BioScope. Biomass specific (net) uptake rates in the BioScope (q_αKG_ = 121.7 μmol/gDW/h and q_glc_ = 633.6 μmol/gDW/h) were comparable (but less accurate) to the uptake rates in the bioreactor. Based on these comparable rates, it was assumed that the q-rates for biomass (μ = 0.103 h^−1^) and CO_2_ production (q_co2_ = 3,132 μmol/gDW/s) were the same for the BioScope and bioreactor experiment. These rate values were used for the flux analysis.
II. Intracellular concentrations are assumed to be constant during the whole BioScope experiment. TCA intermediates show stable trends over the whole duration of the experiment (Figure S3), deviations here can be ascribed to noise in the measurements. The glycolytic intermediates show a concentration drop in the beginning of the experiment, but over time the concentrations increase to the steady-state concentrations again. This drop might be caused by the travel time in the recycle loop and small changes in conditions in the BioScope. These glycolytic concentration changes do not have an impact on the labeling dynamics as glucose is unlabeled.
III. αKG exists in the mitochondria and the cytosol—the respective (amount) fraction is included by two distinct pools: αKG_cyt and αKG_mit. For other metabolites no compartment specific pools were introduced, assuming that these metabolites were fully mitochondrial or cytosolic. The cytosolic fraction of the total αKG pool is described by a parameter (f_αKG) that is included in the parameter estimation for concentration (C) and enrichment (X) data. This is implemented by the following equations:
  $$\begin{aligned} C_{akg} & = C_{akg,cyt} + C_{akg,mit} = C_{akg} *f_{akg,cyt} + C_{akg} *(1 - f_{akg,cyt} ) \\ X_{akg} & = X_{akg,cyt} + X_{akg,mit} = X_{akg} *f_{akg,cyt} + X_{akg } *(1 - f_{akg,cyt} ) \\ \end{aligned}$$
IV. No gluconeogenetic activity: Labeling is introduced via αKG uptake; With no gluconeogenic activity, no labeling will be present in pyruvate and upstream metabolites. Then glycolysis can be lumped into one reaction from G6P to two Pyruvate. The pentose phosphate pathway (PPP) was not included, as this pathway has no influence on labeling patterns under the experimental conditions.
V. Glutamate dehydrogenase was assumed to be solely cytosolic (Table S3).
VI. Biomass formation and CO_2_ formation were assumed to be the only carbon sinks. Biomass formation was described by including a biomass equation consuming 5 biomass precursors (G6P, AcCoA, OAA, Pyr and Glutamate). The biomass equation was derived from^41^ (Table S2).
VII. Intracellular (mitochondrial) AcCoA concentrations were assumed to be 0.25 μmol/gDW based on^32^, whereas OAA concentrations were assumed to be equal to AcCoA concentrations.
VIII. αKG transport between the cytosol and the mitochondria can be facilitated by citrate–αKG^34^ and malate–αKG antiporters^35^. As we assumed that citrate and malate are solely mitochondrial, we did not include these antiporters in our model, and transport is modeled as uniport of αKG. This was necessary as the slow enrichment increase did not allow for higher model complexity.
IX. At t = 0 h, all metabolites were assumed to be in the natural carbon enrichment state (1.1%). Malate enrichment was measured to be below 1.1% for the first 50 s. This was assumed to be caused by an absolute bias in the measurements and was corrected by adding 0.53% to all malate measurements.
X. Bidirectional fluxes were constraint to a maximum exchange rate of 90 mmol/gDW/h.

The estimation of fluxes was performed based on least-squares optimization to fit both (constant) concentration and labeling enrichment measurements. Simulations were run on MATLAB 2018a, using the fmincon optimization function. To calculate standard deviations for the estimated parameters, a Monte-Carlo analysis was performed: 2.5% Gaussian noise was added to the measured enrichment data and simulations were run 100 times to calculate deviation in the estimated parameters. Flux map figures were prepared using the visualization software Omix^42^.

### Ethics approval and consent to participate

Not applicable.

### Consent for publication

Not applicable.

## Supplementary information

Supplementary information.

## Data Availability

The datasets supporting the conclusions of this article are included within the article and its additional files.
