# Supplementary material for: Alpha-ketoglutarate utilization in Saccharomyces cerevisiae: transport, compartmentation and catabolism
Source: Sci Rep. 2020 Jul 30;10:12838. doi: 10.1038/s41598-020-69178-6 (PMC7393084; doi:10.1038/s41598-020-69178-6)
Supplement: Supplementary file 1 — Supplementary information. [file 41598_2020_69178_MOESM1_ESM.pdf]

Alpha-ketoglutarate utilization in *Saccharomyces cerevisiae:* Transport, compartmentation and catabolism

Jinrui Zhang[1], Bastiaan Mees van den Herik[1], Sebastian Aljoscha Wahl[1]

Supplementary Material

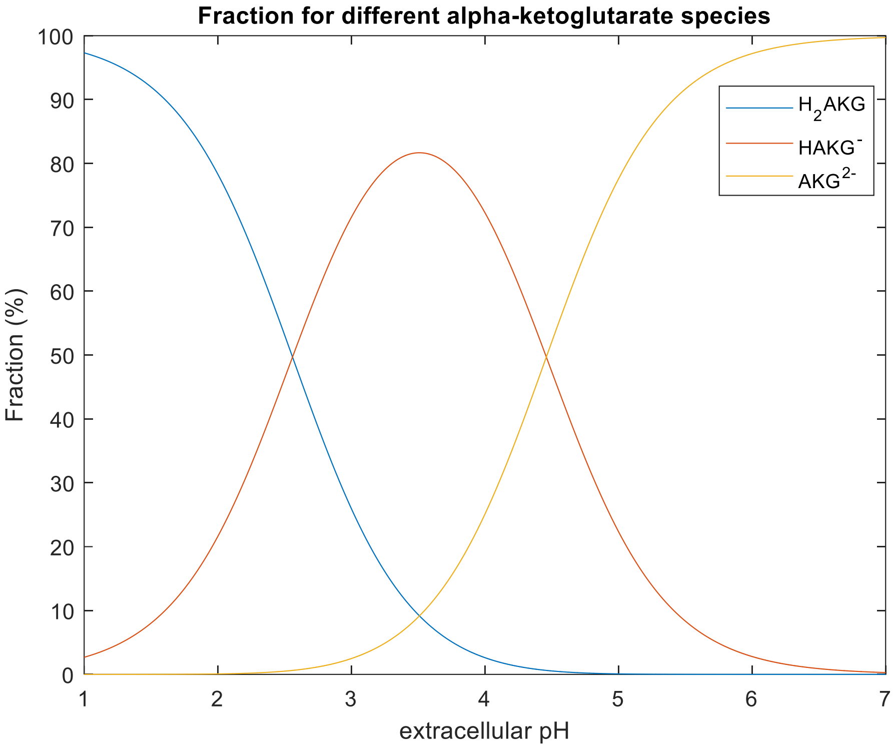

Figure S1: Species distribution of αKG as a function of the extracellular pH.

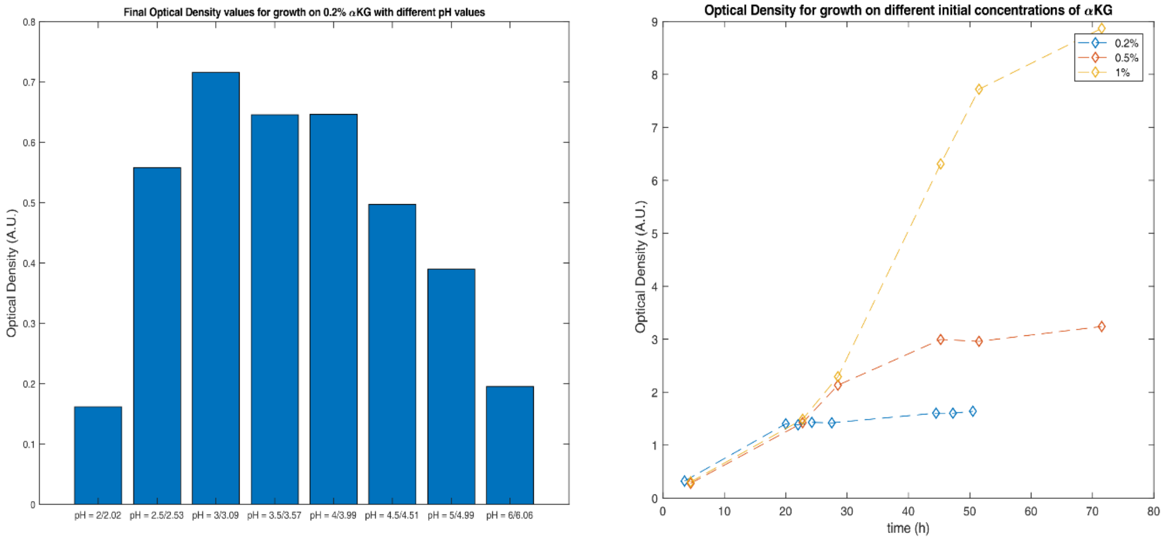

Figure S2: Left: Final OD of batch cultivations at constant αKG concentration at different pH (starting pH/final pH). Right: Biomass concentration (OD) time-course for different αKG starting concentrations at pH=3.

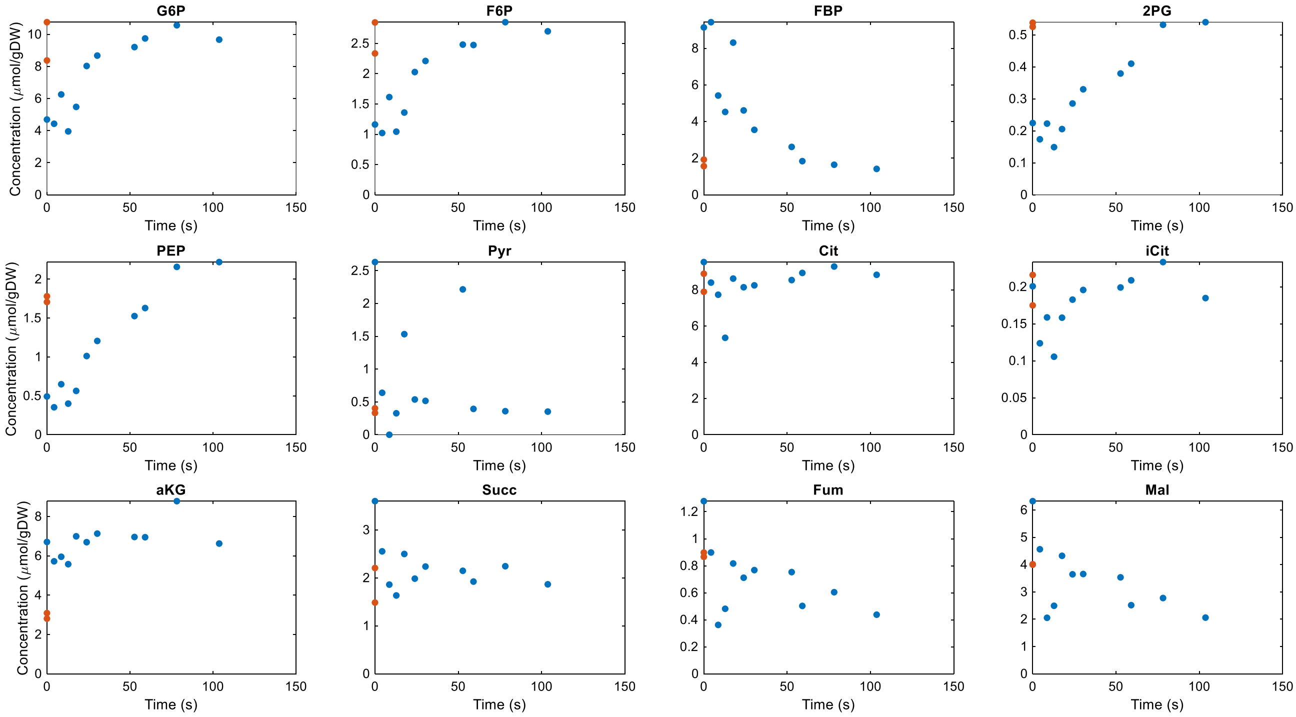

Figure S3: Intracellular concentrations in the bioreactor before and after the BioScope experiment (orange markers) and at the different timepoints in the BioScope (blue markers)

Table S1: Stoichiometric reactions for the metabolic network including the atom transition with each reaction. Unidirectional fluxes use “>” as the reaction arrow while bidirectional fluxes use “<>” as the reaction arrow.

**Reaction**	**Atom transitions**
FeedA: FeedA > GlcEC	#ABCDEF > #ABCDEF
FeedB: FeedB > αKG EC	#ABCDE > #ABCDE
v_upt_Glc: GlcEC > G6P	#ABCDEF > #ABCDEF
v_upt_AKG : αKG_ec > αKG _cyt	#ABCDE > #ABCDE
rUPGly: G6P > Pyr + Pyr	#ABCDEF > #CBA + #DEF
rPDH: Pyr > AcCoA +CO2	#ABC > #AB + #C
rCIT: OAA + AcCoA> Cit	#ABCD + #EF > #ABCDEF
rACO: Cit > iCit	#ABCDEF > #ABCDEF
rIDH: iCit> αKG _mit + CO2	#ABCDEF > #ABCDE + #F
rKGD: αKG _mit > Succ + CO2	#ABCDE > #ABCD + #E
rSDH: Succ > Fum	#ABCD > #ABCD
rFUM: Fum <> Mal	#ABCD > #ABCD
rMDHm: Mal <> OAA	#ABCD > #ABCD
tAKG : αKG _cyt <> αKG _mit	#ABCDE > #ABCDE
rGDH: αKG _cyt <> Glutamate	#ABCDE > #ABCDE
rPyc: Pyr + CO2 > OAA	#ABC + #D > #ABCD
CO2in: CO2_ex > CO2	#A > #A
CO2_sink: CO2 > CO2_out	#A > #A

Table S2: Biomass reaction derived from Lange & Heijnen (2001)

Precursor	Requirement (µmol/gDW)	Requirement (mol/Cmol)
**G6P**	3485	0.0924
**Glutamate**	1105	0.0292
**OAA**	982	0.0259
**Pyruvate**	1381	0.0365
**AcCoA**	2158	0.0570
**CO2**	-936	-0.0247
**NADH**	1338	0.0353
**NADPH**	10098	0.2666

Glutamate Dehydrogenase localization in *S. cerevisiae*.

Three isozymes of the enzyme glutamate dehydrogenase (GDH) are encoded in the *S. cerevisiae* genome; two are NADP-dependent *GDH1* and *GDH3* and one is NAD-dependent *GDH2*. Localization of GDH isozymes is subject of studies and differs per species (DeLuna et al.,2001)). In *S. cerevisiae, GDH1, 2, 3* are confirmed to be active in the cytosol and nucleus by enzymatic assay (Camardella et al., 1976; Perlman & Mahler, 1970) and GFP-fusion (Huh et al., 2003). Localization is still not fully resolved as Sickmann et al. (2003) report mitochondrial presence of *GDH2,3* using proteome analysis.

In the constructed model the localization of all *GDH* isozymes is assumed to be cytosolic and thus a reversible glutamate dehydrogenase reaction is only, and fully, present in the cytosol.

Table S3: Localization of glutamate dehydrogenase.

Source	*GDH1*	*GDH2*	*GDH3*
Perlman & Mahler (1970), Camardella et al. (1976)	Cytosol/ Nucleus *	Cytosol	Cytosol/ Nucleus *
Huh et al. (2003)	Cytosol/Nucleus	Cytosol	-
Sickman et al. (2003)	Cytosol	Mitochondria	Mitochondria

*The presence of *GDH1* and *GDH3* isozymes was unknown at the time

FBA approach

Carbon uptake was fixed to the amount as measured in the experiment. Total uptake was changed to Cmol, which was used to calculate different fractions of carbon introduced by αKG import. Growth rate was fixed to the experimentally measured growth rate (μ=0.103h-1). Glycolysis and αKG import of αKG into the mitochondrion are plotted. These two fluxes give information on the growth modes possible when growing on a mixture of αKG and glucose. For low αKG fractions it is expected that all αKG is used for glutamate biosynthesis in the cytosol, and no transport into the mitochondria for oxidation is present. This leads to the most efficient use of αKG.

At higher fractions αKG part of αKG is used to fully supply glutamate biosynthesis and another part is oxidized in the mitochondria using the TCA cycle. At high αKG fractions, gluconeogenic flux is needed to produce glucose and other glycolytic precursors for biomass formation.

Table S4: Growth modes co-consumption αKG and glucose

**Growth mode**	**αKG fraction (%)**
αKG solely used for glutamate production	9.29
Need for gluconeogenic flux	70.70
Growth no longer possible for experimental growth rate	93.90

In the labelling experiment performed, an αKG /glucose fraction of 12.5% was used. According to the FBA prediction. This will lead to TCA-cycle activity which will results in enrichment of other TCA-cycle intermediates. As predicted by the FBA, only a small percentage of αKG is used for TCA-cycle activity, so unless there is a large exchange between the cytosol and mitochondria there will be a low amount of labelling observed (as is the case in the experimental results).

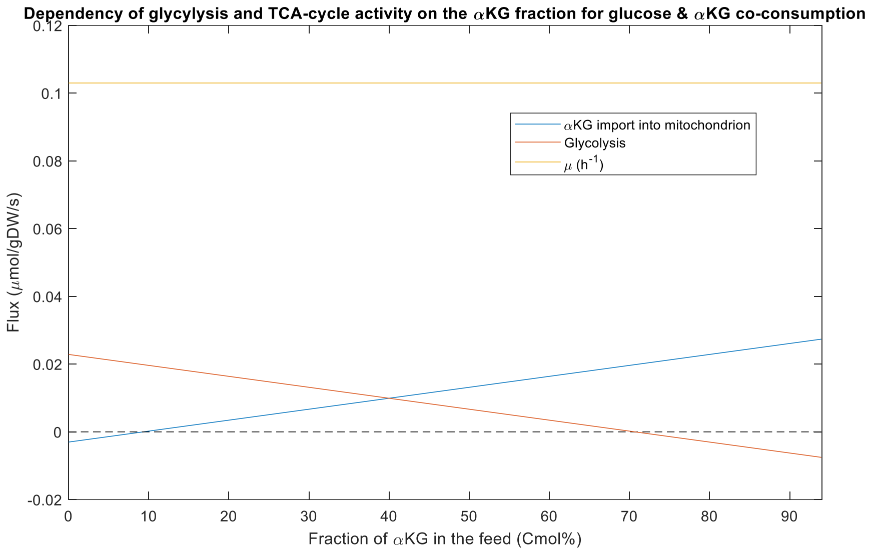

Figure S4: Glycolytic and αKG transport flux as a function of the fraction of carbon origination from αKG for the experimentally obtained growth rate and uptake rates.

TCA-cycle direction

To verify the estimated cytosolic fraction of αKG, it was checked whether the TCA-cycle was still able to be active in the oxidative direction with a mitochondrial concentration of 7.01mM. Equilibrium constants under biochemical standard conditions (K_{eq}) and the reaction quotient (Q) were calculated (Table S5), whereas K_{eq}>Q the reaction is active in the forward direction. These results show that both the reaction towards as the reaction from αKG run in the forward direction, verifying the biochemical validity of the found αKG fraction. This analysis is based on a range of assumptions e.g. CO_2, NAD^+/NADH concentrations, iCit and Succ are present solely in the mitochondria and the volume of the mitochondria is 7% of the total cellular volume. Nonetheless, this analysis shows that the differences between Q and K_{eq} is so large that it can be used to interpret the obtained αKG fraction.

Table S5: Thermodynamically derived direction of mitochondrial production and consumption of αKG.

**Reaction**	**Equilibrium constant (K_{eq})**	**Reaction Quotient (Q)[1]**	**Direction**
isocitrate +NAD^+ -> alpha-ketoglutarate$_{mit}$ + CO_2 + NADH	0.11	0.022	Forward
alpha-ketoglutarate$_{mit}$ + ADP + P_i + NAD^+ -> succinate + CO_2 + ATP + NADH	34000	4.02	Forward

References supplementary material

Camardella, L., Prisco, G. D. I., Garofano, F., & Maria, A. (1976). Purification and properties of nadp-dependent glutamate d e h y d r o g e n a s e from yeast n u c l e a r fractions, *429*, 324–330.

Perlman, S., & Mahler, H. R. (1970). Intracellular Localization of Enzymes in Yeast ' In the course of our studies on the bio- genesis of mitochondria in baker ' s yeast.

Sickmann, A., Wagner, Y., Joppich, C., Meyer, H. E., Perschil, I., Chacinska, A., … Meisinger, C. (2003). The proteome of Saccharomyces cerevisiae mitochondria. *Pnas*, *100*(23), 13207–13212.

Huh, W.K., Falvo, J.V., Gerke, L.C., Carroll, A. S., Howson, R.W., Weissman , J.S., O'Shea, E.K. (2003). Global analysis of protein localization in budding yeast. Nature425, 686–691.
